# Medical Character of Saratoga Springs

**Published:** 1847-07

**Authors:** M. L. North

**Affiliations:** Saratoga


*Medical Character of Saratoga Springs.* By M. L. North, M. D., of Saratoga.—There are two modes of ascertaining the medical character of any therapeutical agent; first, chemical analysis; and second, clinical observation. In considering the first of these modes, namely, the investigations of the chemical qualities of an agent, I copy here the third edition of my Invalid's Guide, the analysis which I obtained in the spring of 1842, from Dr. James R. Chilton, of New York.

One gallon, or 231 cubic inches, contains:

| | |
|---|---|
| Chloride of sodium, - - - - - | grs. 363·829 |
| Carbonate of soda, - - - - | 7·200 |
| Carbonate of lime, - - - - | 86·143 |
| Carbonate of magnesia, - - - | 78·621 |
| Carbonate of iron, - - - - | ·841 |
| Sulphate of soda, - - - | ·651 |
| Iodide of sodium and bromide of potassium, | 5·920 |
| Silica, - - - - - - | ·472 |
| Alumina, - - - - - - | ·321 |
| | Grs. 543·498 |

| | |
|---|---|
| Carbonic acid, - - - | 284·65 |
| Atmospheric air, - - - | 5·41 |
| Cubic inches, - - - | 290·05 |

Although compounded in a region far removed from our observation, it so happens that every article of the twelve, except silica and atmospheric air, have been used in our materia medica, and most of them have long ben familiar articles, yet, there is not a member of that body, who would not perceive from first sight, that the congress water has the following medicinal or therapeutic qualities, viz.: first, laxative or aperient; second, diuretic; third, antacid; fourth, deobstruent; fifth, alterative; and sixth, tonic.

Every one of these qualities is deducible from the analysis; but long before chemistry was applied to these waters, common observation had abundantly established these very identical properties. It is hence very manifest that there is a most striking coincidence between the two modes of ascertaining the medicinal character of these waters. There are, however, two or three properties of these waters which would never have been discovered by *à priori* reasoning.

First. *The augmented effect of the combination*, contrasted with the individual effects of the respective articles. There is, probably, not a member of the convention who has not been surprised at the augmented effect of some of his extempore prescriptions. This effect of combination was fully illustrated and placed on its right basis by Paris in his Pharmacologia, and has been fully considered by subsequent writers on the materia medica. Who would dream, by examining the contents of three or four tumblers of congress water, that they would prove an efficient and immediate cathartic? The principal laxative articles, if not the only ones, contained in one quart, the usual morning potation, are ninety-one grains of table salt, muriate of soda, and twenty grains of carbonate of magnesia. And yet there is scarcely one failure of a cathartic operation in twenty trials, of taking a qaart before breakfast. But who would have inferred it *à priori?*

Besides the effect of combination just considered, I acknowledge that I have been much influenced in reasoning on the *modus operandi* of the congress water, by the experiments of Dr. Beaumont on the stomach of St. Martin. It was there established that if animal broth, veal soup for example, were introduced into his stomach, the gelatin, fibrin, etc., were deposited on the coats of the stomach, while the watery parts were absorbed by the mucous membrane of this viscus before reaching the duodenum. Alcohol, wine, saline solutions and similar substances were absorbed directly from the cavity of the stomach. It is, therefore, unquestionable that not only the magnesia, but the lime and iron which are rendered soluble by the abundance of carbonic acid ever present, nay, all the ingredients, including the carbonic acid, are carried in a few minutes to the thoracic duct, descending vena cava and heart, and floated in the serum to the brain, spinal marrow, viscera, blood vessels and the minutest vessels of the secretory systems.

But, take another view of this same thing. Suppose a quart of the congress water should be carefully evaporated. By looking over the analysis, you see that the various salts that would be left from the evaporation would amount to about three teaspoonfuls, moderately heaped. Suppose these were mixed with molasses and taken before breakfast instead of one quart of the water. Ninety-one grains of table salt are less than many of us take with our breakfast. The twenty grains of magnesia would not be expected to operate, though combined with the salt, if taken in syrup. Combined with these there is a large proportion of carbonate of lime or chalk, and some iron and soda, none of which should we expect to *aid* in the laxative effect. It seems scarcely admissible, therefore, to adopt any other theory of the thorough cathartic operation of these waters than that they pass in the serum, through the general circulation to the colon, and there produce this specific, watery secretion.

At sulphur fountains, invalids have none but solid or semi-solid dejections. It is not possible to have watery stools, however large the quantity of sulphurous waters taken. At our Saratoga springs, the whole cathartic effect is on the colon; and the dejections are without exception, as far as my knowledge extends, of a watery character. The sulphurous elements of the sulphur springs probably enter the blood: but their place of elimination is mainly in the small intestines, producing solid and consistent stools, while the outlet of the saline springs is, as above shown, the colon and rectum. But I must not tax your patience longer on the augmented, cathartic effects of congress water, and will proceed to consider a second quality that would not be expected *à priori*, and that is, *their tonic effect.* Although there is less than one grain of carbonate of iron in a gallon, its free admission to the blood and inner coat of the branches of the blood-vessels, has been supposed to render it more efficient as a tonic than fiftyfold the amount thrown into, and passing through, the alimentary canal. Beside the iron, there is scarcely an article in the congress water, excepting carbonic acid, and possibly carbonate of lime, that can produce the heating and inflammatory movements caused by that fluid. That it is the iron, is rendered still more probable from the fact that the pavilion water, which contains about four times as much iron, and the Putnam fountain, which contains more than seven times as much iron, while their other ingredients are very similar to those of the congress spring, are greatly more injurious in all inflammatory complaints. I will not, however, stay to theorize on the tonic qualities of these waters. Any one who will read the authentic reports of the various mineral springs in Europe and America will be convinced that all, except the simple saline and cathartic springs, are tonic and stimulating. Even your own Clarendon spring, which contains only 8·75 grains of solid matter to the gallon, and no iron, is allowed on all hands to aggravate inflammatory diseases. Is it not, therefore, most certain, that the perfect solution of these otherwise insoluble articles, contained in congress water, and their introduction into the minute vessels of the sanguiferous and secerning systems, is the cause of their augmented operation?

There is one more effect of these mineral waters which is out of all proportion to the sum of their individual forces, and which would not be inferred *á priori*, and that is: thirdly, *their alterative effect.*

To you, sir, who have spent several weeks here this season with such signal blessing, it is probably not unknown that many leave here after spending two to four weeks, using the waters without the least apparent amendment, nay, with great irritation and discomfort, who, soon after reaching home commence convalescence and continue it till an entire cure is effected. If all the visitants to these springs were called upon to express an opinion of the efficacy of these waters six weeks after reaching home it would be much more favorable than on leaving here. There is a spring in Germany to which patients resort with the express understanding that they are to grow worse and worse for four weeks, by means of the perturbations of the waters, when they leave for home, experiencing their convalescence after leaving the spring.

It is true, that a great majority of those who are benefited by the Saratoga waters, convalesce here in some measure. But, the crowning excellence of these waters is the abiding hold the invalids obtain on the amendment they receive here and at home.

But I return to the inquiry: what is there in the Saratoga waters that would warrant the expectation or certainty that a permanent amendment would so signally follow from a course of a few weeks' potation and bathing? By recurring to the list of articles, there are only two medicines with which we are not perfectly familiar, and to these I shall limit my remarks. These are the *iodide of sodium* and *bromide of potassium.* If ever there was a medicine that came into the materia medica through the right door, in that noiseless, unostentatious, but steadily-progressing manner which gives strong promise of permanent favor and employment, it is the hydriodate of potassa. I forbear to *expatiate* on its wide-extended applicability and usefulness. But what unexpected results have occurred from its use? How many cases of asthma, that real opprobrium of the profession, have been relieved or cured? Dr. Mutter's case of lupus, on the nose of an asthmatic patient, calling for use of iodide of potassium: what untold relief has it accidently wrought among the ranks of asthmatics? Many are "as good as cured." Yet there is no rational *modus operandi* announced by the faculty. This article, in its serpentine, sinuous adaptation to the removal of multiform, chronic ailments, hitherto supposed to be perfectly dissimilar, seems to have confounded and pronounced baseless the venerable classification of medicines. Classification! It is a conventionality. It is an artificial bond, formed for covenience. Look at sulphate of zinc and tartrate of antimony. The one entonic, stimulating, building up, even when it vomits, the other, a deadly, depressing agent. Yet, both emetics. Look at iodine, grown in plants that were matured in a living fluid, a fluid that would kill all the vegetables on which we have heretofore experimented, and its nature is as unique as its origin. Who can classify iodine and its compounds? Quinine, what is that like? Does the word *tonic* tell at all its qualities?

But to return to the hydriodate of potassa. I have known a patient at the commencement of a frightful paroxysm of asthma in the night, take thirty grains of this medicine, and in forty minutes be asleep for the night. In the morning he could give no account of it. There was no increased action of bowels, kidneys, blood-vessels, nor any known change of action, except looseness of mucus in the lungs. If he has to repeat these large doses a few nights, he finds a bitter taste in his mouth, showing that the blood is fully impregnated with the article. There may be also slight swelling of the gums, soreness of the teeth, and possibly eruption on the face. These are *all* the evils; the alleged consequences of injury to the absorbent system, never having in my opinion, resulted from the hydriodate, whatever may be said of the iodine itself. Having taken it three and a half years in my own person, as incomparably the best palliative for asthma in doses of all grades, from infinitesimal up to ℥ ij. at once!! and received careful statements from other physicians who have watched its effects, I cannot believe in its injuring the tissues. It is truly an alterative in asthma—that is, "a remedial agent, that restores health to the system, in a gradual, imperceptible manner, without any marked sensations or uncommon evacuations during their operation." In this way it restores health in the multiplied phases of scrofula, and, in brief, in all the diseases resulting from generally-vitiated constitution or abnormal, cachectic action.—*N. Y. Journal.*

